# Multiparametric MRI and [^18^F]Fluorodeoxyglucose Positron Emission Tomography Imaging Is a Potential Prognostic Imaging Biomarker in Recurrent Glioblastoma

**DOI:** 10.3389/fonc.2017.00178

**Published:** 2017-08-18

**Authors:** Comron Hassanzadeh, Yuan James Rao, Anupama Chundury, Jackson Rowe, Maria Rosana Ponisio, Akash Sharma, Michelle Miller-Thomas, Christina I. Tsien, Joseph E. Ippolito

**Affiliations:** ^1^Department of Radiation Oncology, Washington University in St. Louis, St. Louis, MO, United States; ^2^Department of Genetics, Washington University in St. Louis, St. Louis, MO, United States; ^3^Mallinckrodt Institute of Radiology, Washington University in St. Louis, St. Louis, MO, United States; ^4^Department of Radiology, Mayo Clinic Florida, Jacksonville, FL, United States

**Keywords:** radiation, MRI, apparent diffusion coefficient, diffusion, [^18^F]fluorodeoxyglucose-positron emission tomography, glioblastoma, radionecrosis

## Abstract

**Purpose/objectives:**

Multiparametric advanced MR and [^18^F]fluorodeoxyglucose (FDG)-positron emission tomography (PET) imaging may be important biomarkers for prognosis as well for distinguishing recurrent glioblastoma multiforme (GBM) from treatment-related changes.

**Methods/materials:**

We retrospectively evaluated 30 patients treated with chemoradiation for GBM and underwent advanced MR and FDG-PET for confirmation of tumor progression. Multiparametric MRI and FDG-PET imaging metrics were evaluated for their association with 6-month overall (OS) and progression-free survival (PFS) based on pathological, radiographic, and clinical criteria.

**Results:**

17 males and 13 females were treated between 2001 and 2014, and later underwent FDG-PET at suspected recurrence. Baseline FDG-PET and MRI imaging was obtained at a median of 7.5 months [interquartile range (IQR) 3.7–12.4] following completion of chemoradiation. Median follow-up after FDG-PET imaging was 10 months (IQR 7.2–13.0). Receiver-operator characteristic curve analysis identified that lesions characterized by a ratio of the SUV_max_ to the normal contralateral brain (SUV_max_/NB index) >1.5 and mean apparent diffusion coefficient (ADC) value of ≤1,400 × 10^−6^ mm^2^/s correlated with worse 6-month OS and PFS. We defined three patient groups that predicted the probability of tumor progression: SUV_max_/NB index >1.5 and ADC ≤1,400 × 10^−6^ mm^2^/s defined high-risk patients (*n* = 7), SUV_max_/NB index ≤1.5 and ADC >1,400 × 10^−6^ mm^2^/s defined low-risk patients (*n* = 11), and intermediate-risk (*n* = 12) defined the remainder of the patients. Median OS following the time of the FDG-PET scan for the low, intermediate, and high-risk groups were 23.5, 10.5, and 3.8 months (*p* < 0.01). Median PFS were 10.0, 4.4, and 1.9 months (*p* = 0.03). Rates of progression at 6-months in the low, intermediate, and high-risk groups were 36, 67, and 86% (*p* = 0.04).

**Conclusion:**

Recurrent GBM in the molecular era is associated with highly variable outcomes. Multiparametric MR and FDG-PET biomarkers may provide a clinically relevant, non-invasive and cost-effective method of predicting prognosis and improving clinical decision making in the treatment of patients with suspected tumor recurrence.

## Introduction

Although significant advancements in the treatment of glioblastoma multiforme (GBM) have occurred in recent decades, survival outcomes remain poor. The median survival is approximately 12–15 months (1) and tumor progression is inevitable for most patients. In the post-treatment setting, treatment-induced changes can confound definitive identification of tumor recurrence and progression (2, 3). Differentiating tumor progression from post-treatment radiographic changes, including radionecrosis, is vital to provide patients with appropriate salvage therapies to extend life and avoid unnecessary toxicity for those without evidence of disease. The advent of new modalities to detect tumor progression earlier in the surveillance period may translate to improved survival for patients with GBM.

Assessing GBM response to therapy has been a challenging and controversial aspect of post-treatment oncologic management. Current National Comprehensive Cancer Network guidelines for GBM treatment response surveillance recommend conventional brain MRI 2–6 weeks after radiation therapy, followed by MRI every 2–3 months for the next 2–3 years, and less frequent imaging thereafter (4). Although diffusion and perfusion imaging using MRI have demonstrated their ability to differentiate recurrence from radiation necrosis (5–7), there remains no gold standard imaging technique to diagnose recurrent disease in GBM patients. The recently published Response Assessment in Neuro-Oncology Criteria (RANO) criteria improved the accuracy of follow-up imaging; however, identification of tumor progression remains a formidable challenge (8).

In addition to MRI, positron emission tomography (PET) imaging has shown promise in the identification of GBM tumor progression (9–11). PET using [^18^F]fluorodeoxyglucose (FDG-PET) is a well-established imaging technique for evaluation of treatment response in other cancers; however, its use in GBM surveillance remains controversial, especially given high baseline cerebral FDG uptake (12–16). In addition to PET imaging, studies have shown the utility of magnetic resonance spectroscopy (MRS) to predict tumor recurrence (17), and a meta-analysis found that MRS alone had modest diagnostic performance in identifying recurrent tumor (18). Although several studies have investigated the ability of FDG-PET to differentiate radionecrosis from true progression, few have investigated the prognostic implications of integrated FDG-PET and MR parameters on patient outcomes (19).

We hypothesized that a combination of FDG-PET and MR imaging parameters could be used to develop a risk stratification system to better identify patients with GBM recurrence. The objective of this study was to evaluate a cohort of patients with GBM with suspected recurrence and to determine optimal imaging parameters using FDG-PET and multiparametric MRI that could be used to predict outcomes.

## Materials and Methods

### Study Population

We retrospectively identified 30 patients with pathologically confirmed GBM (WHO grade IV) who received treatment consisting of biopsy or surgery, external beam radiation therapy, and concurrent and adjuvant temozolomide (1) between 2001 and 2014; and subsequently developed an enhancing lesion on conventional contrast-enhanced MRI that was presumed to be suspicious, but not definitive, for tumor progression according to the RANO Criteria (8) and review at multidisciplinary tumor board. These patients were initially identified from our institution’s medical record to include all patients who underwent the treatment regimen as described above in addition to undergoing a FDG-PET/CT scan. All patients received a brain FDG-PET/CT scan between 2007 and 2015 to evaluate for treatment effect versus true progression. This retrospective analysis was approved by the Washington University Institutional Review Board.

### MRI and PET Imaging Technique

Baseline MRI and FDG-PET scans were obtained at the time of suspected progression. Twenty-eight of the patients underwent separate 1.5 T MRI (Sonata or Symphony; Siemens, Erlangen, Germany) and hybrid FDG-PET/CT scans (Biograph Duo or LSO-40; Siemens, Erlangen, Germany), while two patients received simultaneous 3 T-MRI/PET acquisition on a hybrid PET/MR scanner (Siemens mMR PET/MR; Siemens, Erlangen, Germany). The patients underwent brain tumor protocol MR imaging (20) before and after intravenous gadolinium contrast. MR sequences included T1-weighted (pre-and post contrast), T2-weighted, fluid attenuation inversion recovery (FLAIR), and diffusion-weighted imaging. Dynamic susceptibility contrast (DSC) perfusion-weighted imaging was performed using a gradient-echo echoplanar imaging sequence during the first pass of gadobenate dimeglumine (MultiHance; Bracco Diagnostics, Princeton, NJ, USA) or gadoversetamide (Optimark; Guerbet, Paris, France) at a dose of 0.1 mmol/kg. Images were acquired at 1 s intervals with intravenous contrast medium injected at a rate of 5 mL/s followed by a 20 mL bolus of saline at the same injection rate beginning on image 10. Perfusion-weighted imaging was processed in all patients for which imaging was acquired and available in the clinical PACS archive. FDG-PET images were obtained beginning 30–67 min (median, 41.5 min) after the administration of 4.4–16.6 mCi FDG (median 10.01 mCi), dosed per the patient’s weight as per standard PET protocol. Non-contrast CT imaging was acquired for attenuation correction and anatomic localization. The median time between the baseline MRI and FDG-PET was 7 days (range, 0–25 days). The MRI and PET images were typically obtained within 3–5 days of each other, while four patients had longer intervals of greater than 2 weeks.

### PET and MR Imaging Analysis

Imaging data were retrospectively post-processed and co-registered based upon cranial anatomy using MIMVista 5 software (MIMVista Corp., Cleveland, OH, USA). Axial T1-weighted gadolinium-enhanced imaging was used to define the region of interest (ROI) by a fellowship-trained neuroradiologist. All patient imaging was processed if clinically acquired and available in the institutional PACS database.

Cerebral blood volume (CBV) maps were calculated from the DSC perfusion data using syngo.Via Neuro Perfusion software (Siemens, Erlangen, Germany) with automated selection of the arterial input function and correction for vascular permeability. CBV were recorded within the ROI identified on contrast-enhanced T1-weighted imaging and in the contralateral normal appearing brain ROI. Subsequently, a ratio of the CBV in the ROI to the contralateral normal CBV was calculated to derive the relative cerebral blood volume (rCBV). Mean apparent diffusion coefficient (ADC) values were analyzed within the ROI.

[^18^F]fluorodeoxyglucose-positron emission tomography ROI were defined by a combination of automatic thresholding using a 40% threshold value of the tumor SUV_max_ as well as further manual editing by the nuclear medicine physician upon review of the corresponding T1-weighted and FLAIR MR imaging for anatomic reference. The threshold value was chosen based on previous publications of high-grade glioma (21) and extracranial tumors (22, 23). To obtain a normalized quantity for FDG uptake in the region of suspected recurrence, a reference contour was placed on a region of normal frontal or parietal lobe normal brain (NB) contralateral to the corresponding lesion ROI. The normal brain ROI was outside of the 20 Gy isodose line in the radiation treatment plan. The normalized quantity was defined as the SUV_max_/NB index. Maximum standard uptake value (SUV_max_), SUV_min_, SUV_mean_, metabolic tumor volume, total lesion glycolysis, and SUV_mean_ of the contralateral normal brain matter reference ROI were recorded from the FDG-PET imaging.

### Follow-up Analysis

Patients typically underwent MR imaging every 2–3 months after completion of chemoradiation therapy and more frequently if clinically indicated. Additional treatment and use of bevacizumab, particularly prior to baseline imaging, was recorded. Additional treatments after baseline imaging (including use of bevacizumab) until last follow-up or death were also recorded.

Patients were retroactively scored on whether they had true progression versus treatment effect in the following manner: patients were scored as having a true progression if they had (i) histopathologic confirmation of residual or recurrent malignant glioma within 6 months of baseline imaging (pathological progression), (ii) two subsequent MRI scans with progressively enlarging tumor within 6 months of baseline imaging according to the RANO criteria (radiologic progression), or (iii) death due to GBM prior to 6 months of follow-up after baseline imaging (clinical progression). Patients were retroactively scored as having treatment effect at the time of baseline imaging if not meeting pathologic, radiologic, or clinical progression criteria within 6 months of the imaging. This composite system has previously been used by other groups to retroactively score for progression versus treatment effect after radiosurgery for brain metastases and, therefore, we adopted it for use in this study (24).

### Statistical Analysis

In addition to true progression versus treatment effect, additional outcome measures for this study were progression-free survival (PFS), and overall survival (OS). Dichotomization of imaging parameters were performed for distinguishing treatment effect versus true progression using receiver-operator characteristic curve (ROC) analysis. The threshold with the maximum sensitivity plus specificity from the ROC analysis was selected. Survival analyses were performed using the Kaplan–Meier method and log-rank statistical test. PFS was defined as the time until the earliest of (i) date of pathologically confirmed progression, (ii) the earlier of the two sequential MRI scans interpreted as progression, or (iii) date of death. OS was defined as the time from the PET scan until death, censoring at last follow-up for those who were alive. These survival estimates were calculated from the time of the FDG-PET scan in the primary analysis and confirmed after survival estimates were re-calculated from the time of surgery. The Cox proportional hazards model was used in univariate analyses of all parameters. χ^2^ or ordinal χ^2^ testing was performed as appropriate. Multivariate analysis was not performed due to small number of patients and a large number of correlated imaging parameters. Significance was considered at a value of *p* < 0.05. All levels of significance were two-sided. SPSS version 22.0 was used for all statistical analyses.

## Results

### Patient Characteristics

**Figure 1:**
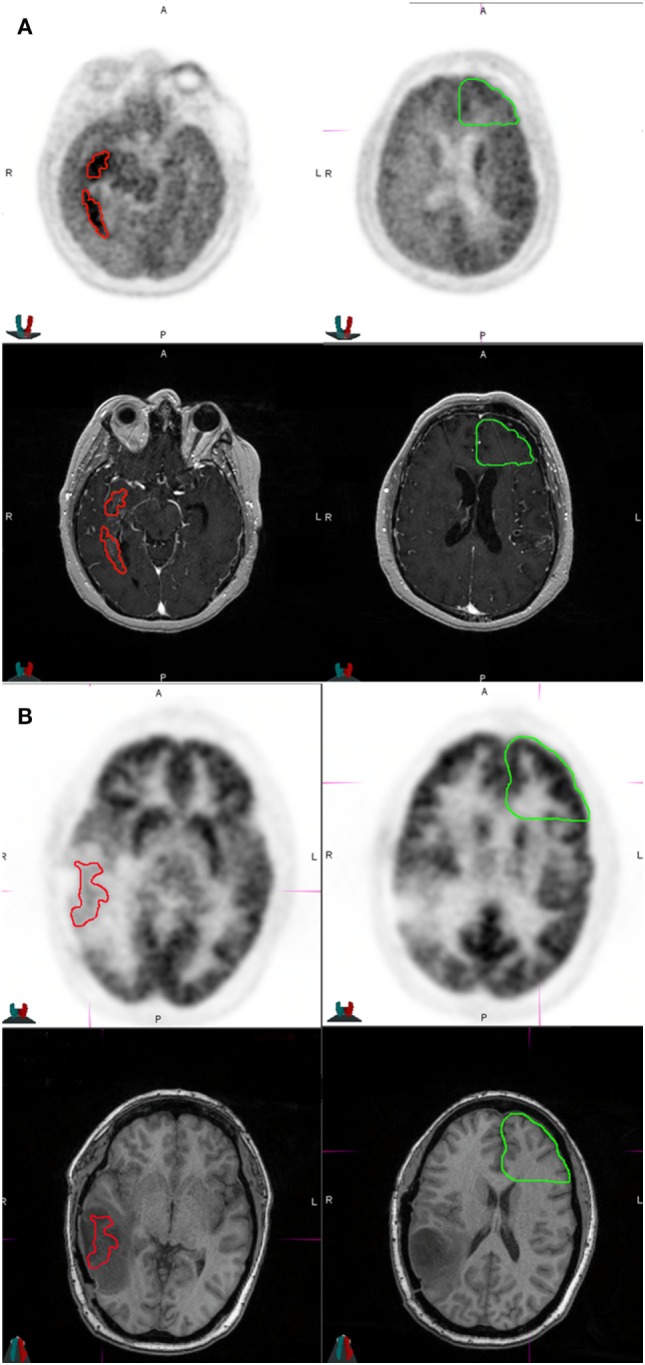
Representative patients in the study who were classified as high risk and low risk at the time of imaging. **(A)** The patient was a 36-year-old male who had MGMT non-methylated, isocitrate dehydrogenase 1 (IDH1) wild-type multifocial glioblastoma multiforme of the right temporal lobe at diagnosis, and received biopsy, radiation therapy to 63 Gy with concurrent temozolomide, followed by adjuvant temozolomide and bevacizumab therapy for 13 months prior to this MRI and [^18^F]fluorodeoxyglucose (FDG)-positron emission tomography (PET) scan. The tumor had a SUV_max_ of 11.64, a SUV_max_/NB ratio of 2.00, and an apparent diffusion coefficient (ADC) of 1,031 × 10^−6^ mm^2^/s. He was classified as high risk and treated with erlotinib. He developed further progression on imaging after 1 month. This patient died of disease at 2 months after this FDG-PET scan. The top two panels show axial slices of the FDG-PET scan at the level of the tumor (left) and normal brain (right). The bottom two panels show axial slices of the T1 contrast image at the level of the tumor (left) and normal brain (right). Red represents tumor region of interest used for deriving imaging parameters. Green represents contralateral uninvolved gray matter for normalizing SUV_max_. **(B)** The patient is a 37-year-old female who had MGMT methylated, IDH1-mutated glioblastoma multiforme of the left temporal lobe treated with gross total resection, radiation therapy to 60 Gy with concurrent temozolomide, followed by adjuvant temozolomide with a tumor treating fields device (Novocure Optune) for 5 months prior to this MRI and FDG-PET scan. The imaging showed an SUV max of 8.6, SUV_max_/NB ratio of 0.60, and an ADC of 1,987 × 10^−6^ mm^2^/s. She was classified as low risk and continued treatment with tumor treating fields device. She did not progress after 6 months and was classified as having treatment effect. She is alive and without progression of disease at last follow-up 13 months after this FDG-PET scan. The top two panels show axial slices of the FDG-PET scan at the level of the tumor (left) and normal brain (right). The bottom two panels show axial slices of the fluid attenuation inversion recovery at the level of the tumor (left) and normal brain (right).

A total of 30 patients were identified using the inclusion criteria. The mean patient age was 52.1 years (range, 21–75) and 17 were male (56.7%). Baseline MRI imaging was acquired at a median time of 7.5 months [range, 0.5–116; interquartile range (IQR), 3.7–12.4] after completion of adjuvant chemoradiation. Baseline PET was obtained at a median of 7 days after baseline MRI imaging. Overall, the median follow-up was 20.8 months (range, 6.6–133; IQR, 15.5–33.4), and the median follow-up after baseline PET scan was 10 months (range, 1.3–32.8 months; IQR, 7.2–13.0). IQRs in addition to range are provided for these intervals as one patient had a very long remission between initial treatment and recurrence, which greatly increased the upper range. O^6^-methylguanine-DNA methyltransferase (MGMT) was methylated in 10 patients, non-methylated in 11 patients, and unknown in 9 patients. Isocitrate dehydrogenase 1 (IDH1) R132 mutation was present in 2 patients, wild type in 21 patients, and unknown in 7 patients. Surgery involved gross total resection in 16 patients, subtotal resection (STR) or near total resection in 12 patients, and biopsy alone in 2 patients. External beam radiation dose was 59.4–63 Gy for all patients. All patients received concurrent and adjuvant temozolomide. Bevacizumab was given as a part of the treatment course in six patients prior to the patient undergoing both PET and MR imaging. Additional patient, tumor, and treatment characteristics are listed in Table 1. Representative ROI used in the analyses are shown in Figure 1.

**Table 1:** Patient and tumor characteristics.

Total number of patients	30	
Median FU after diagnosis (IQR)	20.8 months (15.5–33.4)	
Median FU after PET (IQR)	10.0 months (7.2–13.0)	
Mean age (range)	52.1 years (21–75)	

	***n***	**%**

**Sex**		
Male	17	57
Female	13	43
**Race**		
White	27	90
Black	2	7
Other	1	3
**KPS at diagnosis**		
≤70	10	33
80–100	20	67
**RPA class**		
III	6	20
IV	21	70
V	3	10
**MGMT methylation**		
Non-methylated	11	37
Methylated	10	33
Unknown status	7	30
**IDH1 (R132) mutation**		
Wild type	21	66
Mutated	2	6
Unknown status	7	28
**Initial surgery**		
GTR	16	53
STR/NTR	12	40
Biopsy	2	7
**Previous progression before PET and MRI**		
No (first suspected progression)	22	73
Yes	8	26
**Other treatment before PET and MRI**		
None (other than initial surgery, RT, and TMZ)	10	33
Chemotherapy	18	60
Surgery	2	7
**Use of bevacizumab before PET and MRI**		
No	24	80
Yes	6	20
**Salvage treatment after PET and MRI**		
None	2	7
Chemotherapy	20	67
Radiation	2	7
Surgery	6	20
**Use of bevacizumab after PET and MRI**		
No	10	33
Yes	20	67

*FU, follow-up; KPS, Karnofsky performance score; MGMT, O^6^-methylguanine-DNA methyltransferase; IDH, isocitrate dehydrogenase; GTR, gross total resection; STR, subtotal resection; NTR, near total resection; IQR, interquartile range; PET, positron emission tomography; RPA, recursive partitioning analysis; TMZ, temozolomide*.

### Follow-up

Four patients had pathologic confirmation of progression versus treatment effect within 6 months of baseline imaging. Pathology revealed recurrent tumor in two patients, radiation necrosis in one patient and extensive treatment effect mixed with small amounts of glioma in one patient. Pathology was obtained at a median of 3.1 months after baseline PET scan (range, 1.0–4.3 months). In the remaining 26 patients, based on the follow-up criteria, 15 patients were scored as having true recurrence and 11 patients were scored as treatment effect.

At last follow-up, 21 patients had died with 20 deaths resulting from recurrent tumor and one death from intracranial hemorrhage. Median PFS and OS of the entire cohort, measured from the baseline FDG-PET scan was 4.9 months [95% confidence interval (CI) 1.0–8.7] and 10.7 months (95% CI 7.7–13.7), respectively. The median PFS and OS of the entire cohort, measured from the date of the surgery were 17.9 months (98% CI 15–21) and 23.7 months (95% CI 21.5–26.2), respectively.

### Association of Imaging and Clinical Factors with PFS and OS

Baseline MRI was acquired at a median time of 7.5 months (range, 0.5–116) after completion of adjuvant chemoradiation. Baseline MRI and PET imaging were obtained at suspected first progression in 22 patients and second progression in 8 patients.

Imaging and clinical parameters were analyzed in a univariate Cox proportional hazards model (Table 2). Factors included MGMT promoter methylation, SUV_mean_ of the enhancing lesion, SUV_max_/NB index, rCBV, and mean ADC. Mean ADC was calculated in 28 patients, as there were 2 patients whose images were unable to be processed. rCBV was determined for 14 patients with available perfusion imaging for analysis. An increased SUV_max_/NB index, when analyzed as a continuous variable, was associated with worse PFS with a hazard ratio (HR) of 1.63 per unit increase in index (95% CI 1.10–2.43, *p* = 0.02) and OS with a HR of 4.53 (95% CI 1.10–18.7, *p* = 0.04). Higher mean ADC, analyzed as a continuous variable, trended toward association with improved PFS with a HR of 0.998 per 1 × 10^−6^ mm^2^/s (95% CI 0.997–1.000, *p* = 0.056) and OS with a HR of 0.998 (95% CI 0.997–1.000, *p* = 0.064). MR perfusion parameters including rCBV were not significantly associated with PFS or OS (*p* = 0.78 and *p* = 0.20, respectively). Clinical parameters, including patient age, Karnofsky performance score (KPS), Radiation Therapy Oncology Group (RTOG) recursive partitioning analysis (RPA) class, surgical extent, other treatment before PET scan, and additional treatment after PET scan, were not associated with PFS or OS. Female sex was associated with improved PFS compared to male sex with a HR of 0.40 (95% CI 0.17–0.97, *p* = 0.04) but not overall OS (*p* = 0.12).

**Table 2:** Univariate analyses of PFS and OS.

	UVA for PFS after PET		UVA for OS after PET	
	HR (95% CI)	*p*	HR (95% CI)	*p*
**Sex**				
Male	Reference		Reference	
Female	0.40 (0.17–0.97)	**0.04**	0.48 (0.19–1.20)	0.12
Age	1.01 (0.98–1.04)	0.69	1.01 (0.98–1.05)	0.51
**Race**				
White	Reference		Reference	
Black	1.87 (0.42–8.31)	0.41	2.24 (0.50–10.07)	0.29
Other	2.26 (0.29–17.70)	0.44	2.51 (0.32–19.89)	0.38
**KPS at diagnosis**				
≤70	Reference		Reference	
80–100	0.64 (0.26–1.60)	0.34	0.64 (0.24–1.69)	0.37
**RPA class**				
III	Reference		Reference	
IV	1.98 (0.37–10.37)	0.42	1.90 (0.36–10.04)	0.42
V	1.26 (0.29–5.51)	0.77	0.94 (0.21–4.23)	0.93
**MGMT methylation**				
Non-methylated	Reference		Reference	
Methylated	0.17 (0.05–0.56)	**<0.01**	0.15 (0.03–0.67)	**0.01**
Surgery				
GTR	Reference		Reference	
STR/NTR	1.56 (0.35–7.03)	0.56	0.33 (0.07–1.72)	0.19
Biopsy	0.82 (0.34–1.98)	0.65	0.49 (0.11–2.24)	0.36
**Previous progression before PET**				
No	Reference		Reference	
Yes	1.18 (0.46–3.02)	0.73	1.56 (0.59–4.14)	0.37
**Other treatment before PET**				
None	Reference		Reference	
Chemotherapy	0.75 (0.31–1.81)	0.52	2.86 (0.92–9.04)	0.07
Surgery	0.75 (0.16–3.60)	0.72	1.30 (0.26–6.57)	0.41
**MRI parameters**				
Mean ADC	0.998 (0.997–1.000)	0.056	0.998 (0.997–1.000)	0.064
rCBV	1.42 (0.79–2.56)	0.25	2.19 (0.953–5.03)	0.7
**ADC cut-point**				
≤1,400	Reference		Reference	
>1,400	0.34 (0.12–0.92)	**0.03**	0.29 (0.08–0.99)	**0.048**
**PET parameters**				
SUV_max_	1.04 (0.94–1.16)	0.42	1.07 (0.98–1.19)	0.23
SUV_min_	1.07 (0.80–1.43)	0.66	0.99 (0.68–1.45)	0.99
SUV_mean_	1.15 (1.01–1.30)	**0.03**	1.13 (1.01–1.27)	**0.03**
MTV	0.98 (0.93–1.03)	0.39	1.01 (0.96–1.05)	0.81
TLG	1.00 (0.99–1.01)	0.92	1.003 (0.998–1.008)	0.32
SUV_max_/NB index	1.63 (1.10–2.43)	**0.02**	4.53 (1.10–18.7)	**0.04**
**SUV**_**max**_**/NB index cut-point**				
≤1.5	Reference		Reference	
>1.5	3.48 (1.35–8.98)	**0.01**	4.65 (1.71–12.65)	**<0.01**
**Risk groups**				
Low	Reference		Reference	
Intermediate	2.05 (0.76–5.57)	0.16	1.73 (0.53–5.70)	0.37
High	4.08 (1.33–12.42)	**0.01**	6.41 (1.81–22.75)	**<0.01**
**Salvage treatment after PET**				
None	Reference		Reference	
Chemotherapy	2.48 (0.32–19.31)	0.39	5.30 (0.52–54.2)	0.16
Radiation	0.78 (0.05–12.78)	0.86	1.40 (0.08–15.20)	0.82
Surgery	1.00 (0.11–9.04)	0.98	1.82 (0.18–18.42)	0.61

*MTV, metabolic tumor volume (cubic centimeters); TLG, total lesion glycolysis (cubic centimeters); NB, normal brain; ADC, apparent diffusion coefficient (×10^−6^ mm^2^/s); rCBV, relative cerebral blood volume; PFS, progression-free survival measured from date of FDG-PET scan; OS, overall survival measured from date of FDG-PET scan; GTR, gross total resection; STR/NTR, subtotal resection or near total resection; FDG, [^18^F]fluorodeoxyglucose; PET, positron emission tomography; HR, hazard ratio; KPS, Karnofsky performance score; RPA, recursive partitioning analysis*.

*p-Values in bold denote statistically significant associations*.

### Selection of Imaging Parameters and Generation of Risk Groups

Because the SUV_max_/NB index and ADC had significant effects on patient OS and PFS, we used these imaging parameters to develop a risk stratification system for patients with recurrent GBM. We entered these parameters into an ROC analysis for identification of optimal thresholds to predict 6-month progression or treatment effect. An ADC threshold of 1,400 × 10^−6^ mm^2^/s was identified that had a sensitivity of 76%, specificity of 64%, and area under the curve (AUC) of 0.73 (*p* = 0.04) (Figure S1 in Supplementary Material). The SUV_max_/NB index with an optimal threshold of 1.50 had inadequate ROC performance with a sensitivity of 33%, specificity of 92%, and AUC of 0.55 (*p* = 0.64) (Figure S2 in Supplementary Material). Although not significant in the AUC analysis, the SUV_max_/NB index threshold of 1.50 was retained due to high specificity, and because it was found to be highly correlated to PFS (*p* = 0.01) and OS (*p* < 0.01). Although these individual imaging parameters were not ideal predictors of treatment effect by themselves, we hypothesized that a composite stratification scheme could be developed that leveraged the enhanced sensitivity of ADC and specificity of the SUV_max_/NB index. We used this composite to determine low, intermediate, and high-risk groups for true progression in this patient population.

### Characteristics and Clinical Outcomes of Patients in Risk Groups

Using our thresholds for optimal stratification, the low-risk category (*n* = 11) comprised patients with SUV_max_/NB index ≤1.5 and ADC >1,400 × 10^−6^ mm^2^/s. Conversely, the high-risk category (*n* = 7) comprised patients with SUV_max_/NB index >1.5 and ADC ≤1,400 × 10^6^ mm^2^/s. The intermediate-risk category (*n* = 11) incorporated all other patients.

Intermediate and high-risk patients were more likely to be male compared to low-risk patients (71–75 versus 27%, *p* = 0.047). Patients in the high-risk group were also more likely to be treated with bevacizumab prior to their baseline MRI and PET scan compared to the intermediate and low-risk groups (57 versus 0–18%, *p* = 0.01). In addition, 45% (5 of 11) patients in the low-risk group eventually received additional surgery or radiation after the baseline FDG-PET scan while the remaining 55% (6 of 11) received chemotherapy alone. By contrast, 86% (6 of 7) patients in the high-risk group received chemotherapy alone, and one patient was observed (*p* = 0.03). The groups were balanced in the use of bevacizumab after baseline imaging (*p* = 0.79) (Table S1 in Supplementary Material).

The rates of true progression in the low, intermediate, and high-risk groups were 36, 67, and 86%, respectively; and the frequencies of treatment effect were 64, 33, and 14%, respectively (*p* = 0.04). Of the six patients who received bevacizumab prior to baseline imaging, 100% (4 of 4) of patients classified as high risk also had true progression while 50% (1 of 2) of patients classified as low risk had true progression. Of the 24 patients not previously treated with bevacizumab, the rates of true progression were 33, 67, and 67% in low, intermediate, and high-risk groups, respectively. The above associations in the bevacizumab treated and not-treated groups were not statistically significant due to the diminishing number of patients in subgroups (*p* = 0.11 and *p* = 0.17, respectively) (Table 3).

**Table 3:** Imaging parameters and classification of risk groups.

	All patients		Low risk		Intermediate risk		High risk	
			SUV_max_/NB index ≤1.5 and ADC >1,400		All other patients		SUV_max_/NB index >1.5 and ADC≤1,400	
Number of patients	*n* = 30		*n* = 11		*n* = 12		*n* = 7	
**PET parameters**	**Mean (range)**		**Mean (range)**		**Mean (range)**		**Mean (range)**	
SUV_max_	9.19 (4.55–20.38)		8.12 (4.55–13.81)		7.54 (5.15–11.63)		13.7 (7.14–20.38)	
SUV_min_	3.74 (1.40–7.11)		3.84 (1.40–7.11)		3.73 (2.17–5.15)		3.61 (2.28–5.35)	
SUV_mean_	6.94 (3.37–23.00)		5.95 (0.48–10.46)		5.70 (3.72–8.65)		10.66 (6.25–23.00)	
MTV	7.19 (0.23–43.66)		9.27 (0.48–40.76)		2.95 (0.23–7.21)		11.23 (0.75–43.66)	
TLG	48.76 (1.07–455.33)		49.34 (3.44–219.36)		16.91 (1.07–62.36)		102.45 (4.77–445.33)	
SUV_max_/NB index	1.61 (0.78–6.70)		1.04 (0.60–1.40)		1.12 (0.60–1.50)		1.90 (1.60–2.00)	
**MRI parameters**	**Mean (range)**		**Mean (range)**		**Mean (range)**		**Mean (range)**	
Mean ADC	1,285 (906–1,987)		1,613 (1,457–1,987)		1,100 (906–1,245)		1,085 (974–1,366)	
rCBV	2.89 (1.02–4.66)		2.70 (1.02–4.66)		2.81 (1.49–3.70)		4.66 (4.66–4.66)	
**Clinical outcome**	**Median (95% CI)**		**Median (95% CI)**		**Median (95% CI)**		**Median (95% CI)**	
PFS	4.9 months (1.1–8.7)		10.0 months (2.2–17.8)		4.4 months (0.4–8.3)		1.9 months (1.5–2.3)	
OS	10.7 months (7.7–13.7)		23.5 months (NC)		10.5 months (4.9–16)		3.8 months (3.2–4.4)	
**Tumor determination at 6 months**	***n***	**%**	***n***	**%**	***n***	**%**	***n***	**%**
Treatment effect	12	40	7	64	4	33	1	14
Tumor progression	18	60	4	36	8	67	6	86

*MTV, metabolic tumor volume (cubic centimeters); TLG, total lesion glycolysis (cubic centimeters); NB, normal brain; ADC, apparent diffusion coefficient (×10^−6^ mm^2^/s); rCBV, relative cerebral blood volume; PFS, progression-free survival measured from date of FDG-PET scan; OS, overall survival measured from date of FDG-PET scan; NC, not calculable; FDG, [^18^F]fluorodeoxyglucose; PET, positron emission tomography*.

*Tumor progression is defined as pathological evidence of GBM. Imaging progression of disease on two sequential MRI scans, or death within 6 months after FDG-PET scan. Treatment effect is defined as no pathological evidence of GBM, lack of further enlargement of lesion on imaging, and no death from disease for 6 months after FDG-PET scan*.

**Figure 2:**
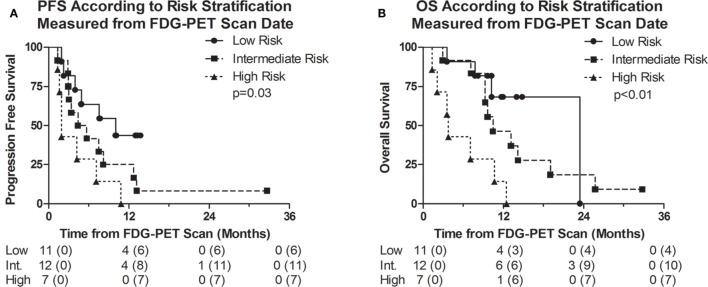
Kaplan–Meier survival curves representing **(A)** progression-free survival (PFS) and **(B)** overall survival (OS) of the entire cohort measured from date of [^18^F]fluorodeoxyglucose (FDG)-positron emission tomography (PET) scan. Numbers below the survival curve represent the number of patients at risk. Numbers in parenthesis represent the number of events prior to the time point.

The median PFS measured from the date of the FDG-PET scan of the low, intermediate, and high-risk groups were 10.0, 4.4, and 1.9 months, respectively (*p* = 0.03); and the median OS measured from the date of the FDG-PET scan were 23.5, 10.5, and 3.8 months, respectively (*p* < 0.01) (Table 3; Figure 2). We also identified that the OS measured from the date of surgery were significantly different among the risk groups at 32.8, 19.0, and 15.4 months (*p* = 0.047), respectively, among the low, intermediate, and high-risk groups. The differences in PFS between the risk groups as measured from the date of surgery were 20.1, 13.0, and 13.3 months, and were not statistically different (*p* = 0.13) (Figure S3 in Supplementary Material).

## Discussion

Currently, there are no available non-invasive imaging methods to easily distinguish between true progression and treatment effect. The utility of radiomics in the post-treatment setting of GBM has vast implications (25–27). Interestingly, radiomics has shown promise in stratifying patients with GBM into short versus long-term survivors (28). Most patients with GBM ultimately develop disease progression, but the problem of determining whether any particular follow-up MRI represents the start of disease progression is a question that remains a significant unmet need in the field. This question is difficult to resolve as most patients who have imaging findings suspicious for disease progression often do not receive further resection or tissue confirmation. In addition, the increased use of bevacizumab and other anti-angiogenic agents has further complicated surveillance imaging of GBM by potentially reducing contrast enhancement and FDG-PET uptake both in viable tumor and radionecrosis (29–31). By contrast, prior studies have suggested that diffusion characteristics of gliomas are not affected by bevacizumab (32). MR perfusion has been shown to be helpful but is not always routinely available and also can be equivocal (5–7, 24, 33–40). The role of radiomics through integrating different imaging parameters may be clinically useful especially in the vulnerable period of post-treatment GBM management.

In this retrospective study, we examined whether the combination of MRI ADC and FDG-PET parameters may be a clinically relevant, and useful tool to improve our ability to predict true tumor progression versus treatment effect in patients with suspected recurrence of GBM. We have identified cutoff values of a SUV_max_/NB index >1.5 and an ADC ≤1,400 × 10^−6^ mm^2^/s that predicted, at suspected recurrence, the probability of true progression. These data suggest the potential for multiparametric MR and FDG-PET imaging as a clinically relevant and easily implemented method that can be routinely used in the clinic.

Several studies have evaluated the utility of post-treatment FDG-PET tumor-to-normal brain ratios in predicting survival in patients with malignant gliomas, including GBM with mixed results (40–43). A study by Barker et al. graded FDG-PET scans by comparing ROI uptake to the contralateral normal cortex in 55 patients with recurrent malignant glioma, 39 of which had GBM (19). The study reported patients with higher PET scores or those with high ratios of uptake in the suspected recurrent lesion had worse survival outcomes than those with lower uptake ratios. In particular, the median survival was 10 months in patients with a higher FDG-PET uptake in the ROI compared to the adjacent cortex, while the median survival was 20 months in patients with a lower ROI FDG-PET score compared to the adjacent cortex. These median survival lengths are similar to the high-risk and intermediate-risk groups in our current study. However, in our study, the incorporation of low ADC into the stratification scheme with high FDG-PET uptake resulted in an even more limited median OS of 3.8 months in high-risk groups. In addition, several previously reported studies have incorporated multiple glioma histologies (33, 34, 39, 44, 45). The limitation in incorporating multiple grades of glioma in a single imaging analysis is highlighted in a study by Yoon et al. which found significant differences in multiple MRI and FDG-PET parameters in patients with low- and high-grade gliomas (35). Therefore, the different imaging profiles of various glioma grades can confound the predictive value of MR and PET imaging specific to GBM. Other studies have confirmed that MR imaging features, including residual T1 enhancing volume (46), ADC (37, 38), and relative CBV (36), have been correlated with survival outcomes in recurrent GBM.

One of the strengths of this small, retrospective study is the integrated model combining MR and FDG-PET imaging parameters. Several advantages are gained by combining MR and FDG-PET modalities in order to determine patient prognosis after suspected recurrence. First, this is an important area of investigation given that MR diffusion imaging along with FDG-PET are readily available in most clinical venues and can be easily incorporated into routine GBM surveillance imaging. By contrast, other promising PET radiotracers such as [^18^F]-DOPA and [^18^F]-FET (10, 24, 47) may not be widely accessible. Second, our study showed the prognostic value of easily attainable and measurable parameters to predict patient survival endpoints in patients with suspected recurrence of GBM. Third, the study population was relatively homogenous with respect to GBM histology, the treatment received prior to PET imaging, and the reproducibility of the technique used to analyze MR and PET imaging.

Prior studies have shown that MGMT promoter methylation status, RPA, and possibly female sex are prognosticators for survival in GBM patients (48–50). Our study confirmed that MGMT methylation status and female sex were associated with better PFS on univariate analysis. However, we did not observe an association between the RTOG RPA class and OS. In addition, we did not identify a correlation between age, KPS, or the extent of resection with PFS or OS. These observations are likely attributable to the small sample size of our study. Interestingly, all patients who were classified as high risk that were treated with bevacizumab prior to the scans had true recurrence of GBM.

There are also several limitations in our study approach. First, the study population is relatively small and derived from a retrospective single-institution review. Therefore, our findings need to be validated using a larger dataset in a prospective fashion. In addition, significant heterogeneity was present in the salvage therapies received by our patients after first recurrence and some of the patients had prior progression events. Also, we included both patients who received total and STRs that may have confounded times to progression. The study also did not have a multivariate analysis to assess potential confounders due to the small cohort of patients. Association between MR and PET imaging parameters and survival outcomes was significant regardless of the salvage regimen administered. True tumor progression at the time of imaging was retroactively scored from radiographic or clinical progression after further follow-up in most patients. Pathology results from surgery or biopsy was also available in only a subset of patients.

## Conclusion

We assessed several MR and FDG-PET parameters and their association with progression and survival outcomes in patients with suspected recurrence of GBM on follow-up MRI scan. Our analysis indicates that integrated MR and PET imaging analyses may be an important clinically relevant tool to improve our ability to distinguish true tumor progression and treatment effect. These results require further confirmation in a larger, prospective study.

## Ethics Statement

This retrospective study was reviewed and approved by the Washington University in St. Louis Institutional Review Board.

## Author Contributions

CH and YR are co-first authors and contributed equally to this work. CH, YR, MP, AS, MM-T, CT, and JI contributed to project conception and planning. CH, YR, AC, JR, MP, MM-T, CT, and JI contributed to data collection and analysis. CT and JI were senior authors and had project oversight.

## Conflict of Interest Statement

The authors declare that the research was conducted in the absence of any commercial or financial relationships that could be construed as a potential conflict of interest.

## Supplementary Material

Figure S1 Receiver-operator characteristic curve of mean apparent diffusion coefficient (ADC) in dichotomizing progression versus radionecrosis.

Figure S2 Receiver-operator characteristic curve of SUV_max_/NB ratio in dichotomizing progression versus radionecrosis.

Figure S3 Kaplan–Meier survival curves representing (A) progression-free survival (PFS) and (B) Overall Survival (OS) of the entire cohort measured from date of surgery.
