# Supplementary material for: Multiparametric MRI and [18F]Fluorodeoxyglucose Positron Emission Tomography Imaging Is a Potential Prognostic Imaging Biomarker in Recurrent Glioblastoma
Source: Front Oncol. 2017 Aug 18;7:178. doi: 10.3389/fonc.2017.00178 (PMC5563320; doi:10.3389/fonc.2017.00178)
Supplement: Supplementary file 1 [file table_1.docx]

**Supplemental Table E1:** Association of Patient and Tumor Factors with Imaging Risk Groups

|  | **Low Risk** | | **Intermediate Risk** | | **High Risk** | | **p** |
| --- | --- | --- | --- | --- | --- | --- | --- |
|  | **n** | **%** | **n** | **%** | **n** | **%** |  |
| **Sex** |  |  |  |  |  |  |  |
| Male | 3 | 27% | 9 | 75% | 5 | 71% |  |
| Female | 8 | 73% | 3 | 25% | 2 | 29% | **0.047** |
| **Race** |  |  |  |  |  |  |  |
| White | 11 | 100% | 11 | 92% | 5 | 71% |  |
| Black | 0 | 0% | 0 | 0% | 2 | 29% |  |
| Other | 0 | 0% | 1 | 8% | 0 | 0% | 0.08 |
| **KPS at Diagnosis** |  |  |  |  |  |  |  |
| ≤ 70 | 8 | 73% | 8 | 67% | 4 | 57% |  |
| 80-100 | 3 | 27% | 4 | 33% | 3 | 43% | 0.79 |
| **RPA Class** |  |  |  |  |  |  |  |
| III | 1 | 9% | 2 | 17% | 3 | 43% |  |
| IV | 10 | 91% | 7 | 58% | 4 | 57% |  |
| V | 0 | 0% | 3 | 25% | 0 | 0% | 0.09 |
| **MGMT Methylation** |  |  |  |  |  |  |  |
| Nonmethylated | 3 | 27% | 4 | 33% | 4 | 57% |  |
| Methylated | 6 | 55% | 3 | 25% | 1 | 14% |  |
| Unknown Status | 2 | 18% | 5 | 42% | 2 | 29% | 0.23 |
| **IDH1 (R132) Mutation** |  |  |  |  |  |  |  |
| Wild Type | 7 | 64% | 8 | 67% | 4 | 57% |  |
| Mutated | 1 | 9% | 1 | 8% | 0 | 0% |  |
| Unknown Status | 3 | 27% | 3 | 25% | 3 | 43% | 0.77 |
| **Initial Surgery** |  |  |  |  |  |  |  |
| GTR | 7 | 64% | 7 | 58% | 2 | 29% |  |
| STR/NTR | 4 | 37% | 5 | 42% | 3 | 43% |  |
| Biopsy | 0 | 0% | 0 | 0% | 2 | 29% | 0.051 |
| **Other Treatment Before PET and MRI** |  |  |  |  |  |  |  |
| None | 8 | 73% | 12 | 100% | 3 | 43% |  |
| Chemotherapy | 1 | 9% | 0 | 0% | 4 | 57% |  |
| Surgery | 2 | 18% | 0 | 0% | 0 | 0% | **<0.01** |
| **Use of Bevacizumab Before PET and MRI** |  |  |  |  |  |  |  |
| No | 9 | 82% | 12 | 100% | 3 | 43% |  |
| Yes | 2 | 18% | 0 | 0% | 4 | 57% | **0.01** |
| **Salvage Treatment After PET and MRI** |  |  |  |  |  |  |  |
| None | 0 | 0% | 1 | 8% | 1 | 14% |  |
| Chemotherapy | 6 | 55% | 8 | 67% | 6 | 86% |  |
| Radiation | 1 | 9% | 1 | 8% | 0 | 0% |  |
| Surgery | 4 | 34% | 2 | 17% | 0 | 0% | **0.03** |
| **Use of Bevacizumab After PET and MRI** |  |  |  |  |  |  |  |
| No | 3 | 27% | 4 | 33% | 3 | 43% |  |
| Yes | 8 | 73% | 8 | 67% | 4 | 57% | 0.79 |
