# Supplementary figures and images for: Multiparametric MRI and [18F]Fluorodeoxyglucose Positron Emission Tomography Imaging Is a Potential Prognostic Imaging Biomarker in Recurrent Glioblastoma
Source: Front Oncol. 2017 Aug 18;7:178. doi: 10.3389/fonc.2017.00178 (PMC5563320; doi:10.3389/fonc.2017.00178)

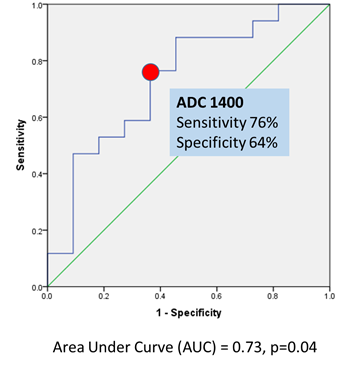

Supplement: Figure S1 — Receiver-operator characteristic curve of mean apparent diffusion coefficient (ADC) in dichotomizing progression versus radionecrosis. [file image_1.tif]

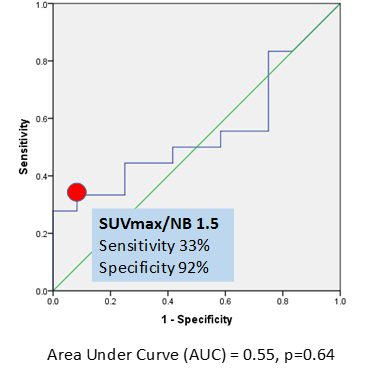

Supplement: Figure S2 — Receiver-operator characteristic curve of SUVmax/NB ratio in dichotomizing progression versus radionecrosis. [file image_2.tif]

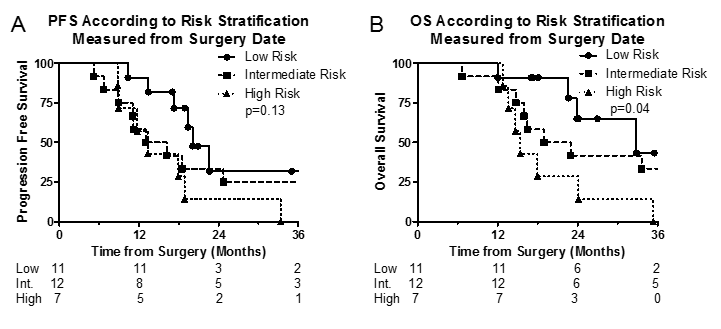

Supplement: Figure S3 — Kaplan–Meier survival curves representing (A) progression-free survival (PFS) and (B) Overall Survival (OS) of the entire cohort measured from date of surgery. [file image_3.tif]
